# The mitochondria-targeted anti-oxidant MitoQ decreases ischemia-reperfusion injury in a murine syngeneic heart transplant model

**DOI:** 10.1016/j.healun.2015.05.007

**Published:** 2015-11

**Authors:** Anna J. Dare, Angela Logan, Tracy A. Prime, Sebastian Rogatti, Martin Goddard, Eleanor M. Bolton, J. Andrew Bradley, Gavin J. Pettigrew, Michael P. Murphy, Kourosh Saeb-Parsy

**Affiliations:** a Medical Research Council Mitochondrial Biology Unit, University of Cambridge and the National Institute for Health Research Cambridge Biomedical Research Centre, Cambridge, United Kingdom; b Papworth Hospital National Health Service Foundation Trust, Papworth Everard, Cambridge, United Kingdom; c Department of Surgery, University of Cambridge, and the National Institute for Health Research Cambridge Biomedical Research Centre, Cambridge, United Kingdom

**Keywords:** ischemia, reperfusion, transplantation, mitochondria-targeted anti-oxidants, MitoQ

## Abstract

**Background:**

Free radical production and mitochondrial dysfunction during cardiac graft reperfusion is a major factor in post-transplant ischemia-reperfusion (IR) injury, an important underlying cause of primary graft dysfunction. We therefore assessed the efficacy of the mitochondria-targeted anti-oxidant MitoQ in reducing IR injury in a murine heterotopic cardiac transplant model.

**Methods:**

Hearts from C57BL/6 donor mice were flushed with storage solution alone, solution containing the anti-oxidant MitoQ, or solution containing the non–anti-oxidant decyltriphenylphosphonium control and exposed to short (30 minutes) or prolonged (4 hour) cold preservation before transplantation. Grafts were transplanted into C57BL/6 recipients and analyzed for mitochondrial reactive oxygen species production, oxidative damage, serum troponin, beating score, and inflammatory markers 120 minutes or 24 hours post-transplant.

**Results:**

MitoQ was taken up by the heart during cold storage. Prolonged cold preservation of donor hearts before IR increased IR injury (troponin I, beating score) and mitochondrial reactive oxygen species, mitochondrial DNA damage, protein carbonyls, and pro-inflammatory cytokine release 24 hours after transplant. Administration of MitoQ to the donor heart in the storage solution protected against this IR injury by blocking graft oxidative damage and dampening the early pro-inflammatory response in the recipient.

**Conclusions:**

IR after heart transplantation results in mitochondrial oxidative damage that is potentiated by cold ischemia. Supplementing donor graft perfusion with the anti-oxidant MitoQ before transplantation should be studied further to reduce IR-related free radical production, the innate immune response to IR injury, and subsequent donor cardiac injury.

Transplantation is the treatment of choice for patients with cardiac failure in whom medical therapy has failed. Although up to 50,000 people are candidates for heart transplantation each year worldwide, only approximately 5,000 adult patients receive a heart transplant.^1^ This mismatch is due to the limited availability of donor hearts that are likely to function adequately after donation, preservation, and reperfusion at transplantation. Therefore, reducing damage to heart grafts at all stages of transplantation is an important goal that should improve outcomes for the recipients of currently acceptable grafts.

Ischemia-reperfusion (IR) injury is an inevitable consequence of organ transplantation and is a key factor in early graft dysfunction and primary graft failure (PGF) in the heart, and the associated tissue damage can lead to induction of an immune response. Prolonged ischemic time is strongly associated with delayed graft function and PGF.^2^, ^3^, ^4^ For cardiac transplantation in patients, the risk of PGF increases 43% for every hour of additional cold ischemia beyond 4 hours.^5^ Prolonged ischemic time beyond 3 hours also linearly increases the 1-year mortality risk in recipients.^1^ In addition, IR injury in recipients may enhance allograft immunogenicity,^6^ increasing the risk of acute allograft rejection and contributing to chronic rejection.^7^ Therefore, minimizing IR injury could improve graft function and survival, decrease the immune response, and may also allow safe extension of cold preservation times.

Even if ischemic tissue is reperfused before significant cell death occurs, reperfusion itself causes extensive oxidative damage initiated by free radical production from the mitochondrial respiratory chain.^8^, ^9^, ^10^ Calcium accumulation within mitochondria during ischemia in conjunction with reactive oxygen species (ROS) activates the mitochondrial permeability transition pore, resulting in cell death.^11^, ^12^ Mitochondrial damage can also initiate an innate immune response through inflammasome activation by mitochondrial ROS^13^ and damaged mitochondrial components such as oxidized mitochondrial DNA (mtDNA).^14^

Conventional anti-oxidants have not previously been successful in preventing mitochondrial oxidative damage in clinical cardiac IR injury,^15^, ^16^ in large part because they are not taken up into mitochondria. To address this, we developed mitochondria-targeted anti-oxidants.^17^, ^18^ The lead compound, MitoQ, consists of a targeting lipophilic triphenyl phosphonium (TPP) cation linked to a ubiquinone moiety.^18^ The TPP cation can pass directly through biologic membranes, allowing rapid uptake into cells and several-hundredfold accumulation within the mitochondrial matrix, driven by the mitochondrial membrane potential.^15^, ^16^, ^17^, ^18^, ^19^ Here, the ubiquinone is recycled by complex II to the active anti-oxidant ubiquinol that prevents oxidative damage (Figure 1A).^17^, ^18^

**Figure 1:**
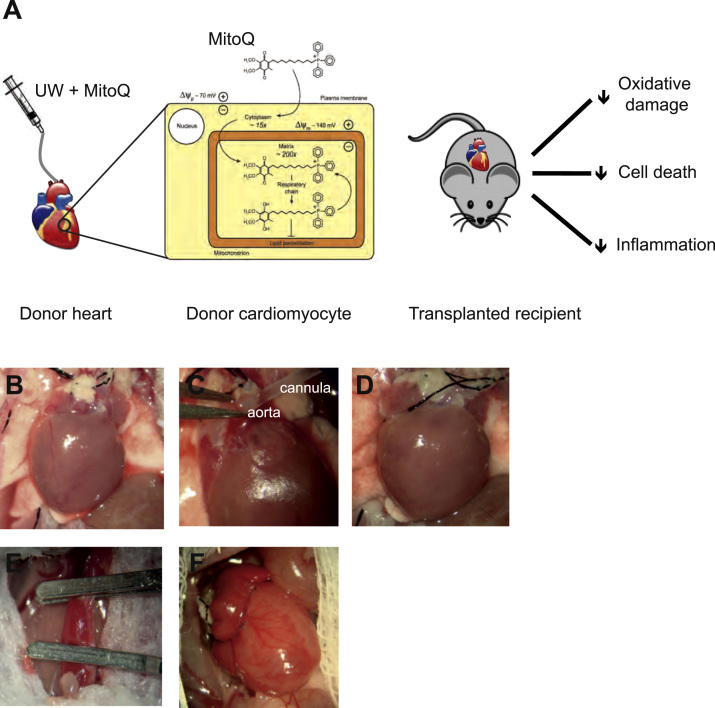
Experimental system. (A) MitoQ uptake and protection during transplantation. Donor hearts were flushed with preservation solution and then stored at 4°C. MitoQ was taken up into the donor heart, accumulating several hundredfold within mitochondria. After cold preservation, the heart was transplanted into the abdomen of the recipient and allowed to reperfuse. (B) The donor heart was removed from the donor. (C and D) The heart was flushed to clear the vasculature of blood and was then placed in preservation solution. (E) The heart was transplanted heterotopically into the abdomen of the anesthetized recipient. A midline abdominal incision in the recipient was made and the inferior vena cava and were aorta exposed and clamped proximally and distally to allow bloodless vascular anastomoses. The donor heart was grafted to the recipient vessels as follows: donor pulmonary artery to recipient inferior vena cava and donor aorta to recipient aorta. (F) The clamps were removed and the graft was revascularized.

MitoQ protects against IR injury in vivo in experimental animal models of myocardial infarction,^20^ hepatic IR injury,^21^ and in renal proximal tubule cells after hypothermia.^22^ In addition, oral MitoQ has demonstrated long-term safety in 2 Phase II clinical trials,^23^, ^24^ although these trials were in diseases with a putative role for oxidative stress in their etiology and were not associated with cardiac reperfusion injury. Here we show that MitoQ administered to the donor heart during cold storage in a syngeneic mouse model of heterotopic cardiac transplantation (Figure 1B–F) protects against oxidative damage and leads to enhanced recovery of the graft upon reperfusion.

## Methods

The experiments in this study were approved by the UK Home Office.

### Heterotopic cardiac transplantation

Male C57BL/6 mice and female Wistar rats were from Charles River Laboratories (Margate, United Kingdom) and were maintained in specific pathogen-free facilities with ad lib food and water. Donor hearts from C57BL/6 mice underwent retrograde flushing in situ with 500 μl Soltran (Baxter Healthcare) cold preservation solution, with or without 50 μmol/l MitoQ, via a 2F cannula (Solomon Scientific) in the ascending aorta. The composition of Soltran is potassium citrate (0.86% w/v), sodium citrate (0.82% w/v), mannitol (3.38% w/v), and magnesium sulphate (1.0% w/v).

Perfusion pressure was applied manually via a syringe (not measured) with a constant perfusion rate of approximately 500 μ1 in 30 seconds. Flushing was visually confirmed under microscopy by filling of the coronary vessels. Hearts were then stored in SPS-1 (University of Wisconsin) solution (Organ Recovery Systems), with or without 50 μmol/l MitoQ or decyl-TPP (dTPP), at 4°C for 30 minutes or 4 hours before transplantation. The composition of SPS-1 is pentafraction (50 g/l), lactobionic acid as lactone (35.83 g/l), potassium phosphate monobasic (3.4 g/l), magnesium sulfate heptahydrate (1.23 g/l), raffinose pentahydrate (17.83 g/l), adenosine (1.34 g/l), allopurinol (0.136 g/l), glutathione (0.922 g/l), and potassium hydroxide (5.61 g/l), pH 7.4. The 50 μmol/l concentration was chosen based on previous dose-response work in small-animal solid organ perfusion systems.^22^

Heart grafts were transplanted into the abdomens of C57BL/6 recipients as previously described (Figure 1B–F)^25^ and were assessed histopathologically at 24 hours by a cardiac pathologist who was blinded to the study groups. Cardiac damage was quantified after 24 hours as plasma troponin I using enzyme-linked immunosorbent assay (Kamiya Biomedical, Seattle, WA). Graft function was assessed visually by observers blinded to the treatment groups at 24 hours using the Stanford Cardiac Surgery Laboratory beat score,^26^ ranging from 0 (no contraction) to 4 (strong regular contraction of both ventricles). The beat score was used as a surrogate marker of graft function.

### Uptake of MitoQ

The uptake of MitoQ and the control compound dTPP was investigated using previously described methods for quantifying uptake into isolated mitochondria using [^3^H]-methyl-TPP and scintillation counting^27^ and into tissues using liquid chromatography and tandem mass spectrometry. For the latter, donor hearts were briefly rinsed in saline to remove MitoQ from the organ surface (see Supplementary Methods, available on the jhltonline.org Web site).

### In situ measurement of mitochondrial ROS

Changes in mitochondrial H_2_O_2_ and peroxynitrite in the heart graft after transplantation were estimated from the conversion of the ratiometric probe MitoB to MitoP.^28^, ^29^ Briefly, 25 nmol MitoB in 100 μl saline was administered via tail vein injection to the recipient 22 hours after transplant. The heart graft was removed after 2 hours, flash frozen in liquid nitrogen, and stored at –80°C until the heart tissue samples (50 mg wet weight) were homogenized, spiked with deuterated internal standards (100 pmol *d*_*15*_*-*MitoB and 50 pmol *d*_*15*_*-*MitoP), and the MitoB and its product MitoP were extracted. The MitoB and MitoP tissue concentrations were determined by liquid chromatography and tandem mass spectrometry.^28^, ^29^

### Determination of oxidative damage to mtDNA and proteins

To assess mtDNA damage, total DNA was isolated from frozen heart graft tissue (~20 mg wet weight) using the Qiagen DNeasy Tissue Kit (Qiagen) and quantified using the PicoGreen dsDNA Assay Kit (Invitrogen). Damage to mtDNA was then assessed using a quantitative polymerase chain reaction method,^30^ based on damage to DNA decreasing amplification of a long mtDNA target sequence (~10 kb) relative to a short target sequence (>200 bp) that controls for mtDNA copy number (see Supplementary Methods, available on the jhltonline.org Web site.). Total protein carbonyl concentration in heart tissue was determined by enzyme-linked immunosorbent assay using the BioCell PC test kit (Biocell Corp, Auckland, New Zealand).^31^

### Inflammatory markers

Serum cytokines (chemokine [C-X-C motif] ligand 1 [CXCL-1], tumor necrosis factor-α, interleukin [IL]-1β, IL-6, and IL-10) were measured by multiplexed electrochemical luminescence immunoassay on the MesoScale Discovery Sector 6000 analyser (Gaithersburg, MD). Samples were analyzed using the MSD 7-plex mouse proinflammatory cytokine high-sensitivity kit (MesoScale Discovery).

### Statistics

Data are presented as means ± standard error of the mean, unless otherwise stated. Statistical analysis was performed with GraphPad Prism 5.0 software (GraphPad Software Inc, La Jolla, CA) using the Student’s 2-tailed unpaired *t*-test. Where several conditions were compared simultaneously, 1-way analysis of variance was used with the Bonferroni correction for multiple comparisons. Data were analyzed assuming normal distribution and were not assessed for normality of distribution. *P*-values < 0.05 were considered statistically significant.

## Results

### MitoQ accumulates in donor cardiac tissue at 4°C

**Figure 2:**
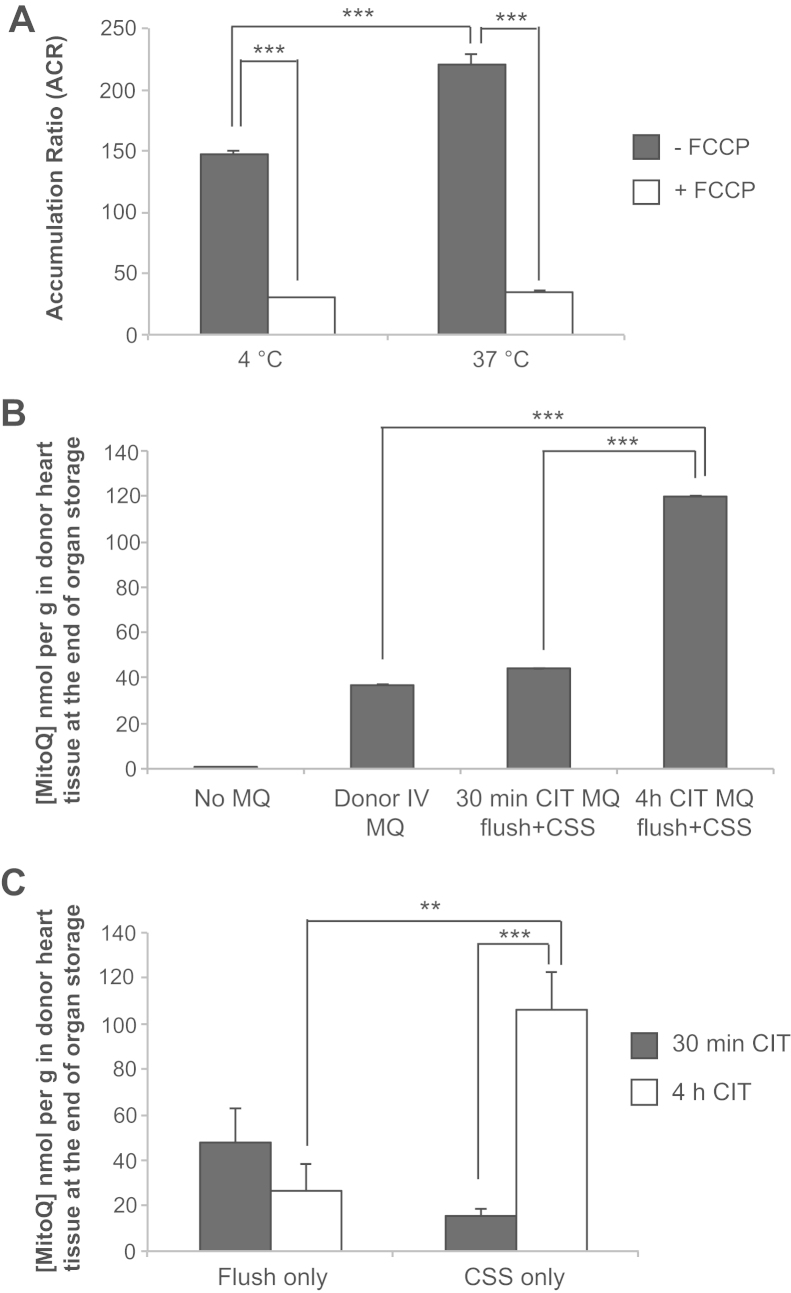
Mitochondria-targeted compounds can be delivered during hypothermic organ preservation. (A) Uptake of [^3^H]-methyltriphenylphosphonium (mTPP) into isolated mitochondria under hypothermic and normothermic conditions, with or without carbonylcyanide *p*-trifluoromethoxy phenylhydrazone (FCCP; *n* = 3). The accumulation ratio (ACR) was calculated as the amount of mTPP in the mitochondria (nmol/mg protein) divided by that in the supernatant (nmol/µ1) after pelleting the mitochondria. (B) Loading of the heart graft before transplantation with MitoQ was assessed by measuring tissue levels by liquid chromatography tandem mass spectrometry (LC-MS/MS). MitoQ was administered by intravenous injection into the donor mouse 15 minutes before organ procurement or during flushing and short (30 minutes) or prolonged (4 hours) cold ischemia time (CIT) preservation of the donor hearts (*n* = 3–4). CSS, cold static storage. (C) To understand MitoQ uptake into the donor heart, hearts were flushed with MitoQ and stored in standard CSS solution or were flushed with standard solution and stored in CSS solution supplemented with MitoQ for 30 minutes or 4 hours (*n* = 3) **p < 0.01, ***p < 0.001 determined by 1-way analysis of variance. Data are mean ± standard error of the mean.

Our goal was to administer MitoQ to donor hearts during in situ flushing and cold preservation to determine whether this decreased IR injury by preventing mitochondrial oxidative damage upon reperfusion (Figure 1). Mitochondrial uptake of MitoQ is driven by the mitochondrial membrane potential, which in ischemic tissues is thought to be maintained by the F_o_F_1_-adenosine triphosphate synthase acting as an adenosine triphosphate hydrolyzing proton pump. There was significant uptake of [^3^H]-methyl-TPP by isolated rat liver mitochondria at 4°C, and abolishing the membrane potential with the mitochondrial uncoupler carbonyl cyanide *p*-trifluoromethoxy phenylhydrazone prevented this uptake (Figure 2A). Therefore even at 4°C, mitochondria sustain a membrane potential that should drive MitoQ uptake within hearts.

We next determined how best to load MitoQ into the heart during cold storage, assessing uptake of MitoQ by measuring its levels in the heart by mass spectrometry (Figure 2B and C). Because MitoQ is taken up rapidly into mitochondria within the heart after intravenous administration,^32^, ^33^ we first measured whether MitoQ was retained by the heart during cold storage after an intravenous injection (2 mg MitoQ/kg) to the donor mouse 15 minutes before removing the heart. After 4 hours of cold preservation, the heart MitoQ content was significant (37 ± 24 pmol/mg wet weight), indicating that MitoQ is retained within the heart during cold storage.

In a clinical setting, systemic administration of MitoQ would affect all organs and controlled delivery in an unstable or hypotensive donor is challenging. We therefore administered MitoQ (50 μmol/l) directly to the donor heart through a cannula placed in the ascending aorta after rapid exsanguination of the mouse (Figure 2B). The heart was then stored in cold MitoQ-supplemented preservation solution, resulting in further uptake of MitoQ into the heart during storage (Figure 2B). In contrast, for hearts flushed with MitoQ and stored in preservation solution without MitoQ, the tissue MitoQ concentration decreased, presumably due to leaching over time (Figure 2C). Therefore, MitoQ can be administered to the donor heart in the preservation solution during flushing and is taken up and retained by the heart. The MitoQ tissue levels achieved by 50 μmol/l MitoQ in the flush and preservation solution were 50 to 120 nmol MitoQ/g wet weight, significantly greater than levels in the heart after oral delivery that are cardioprotective (20–110 pmol/g wet weight^20^, ^34^).

### MitoQ protects against IR injury

**Figure 3:**
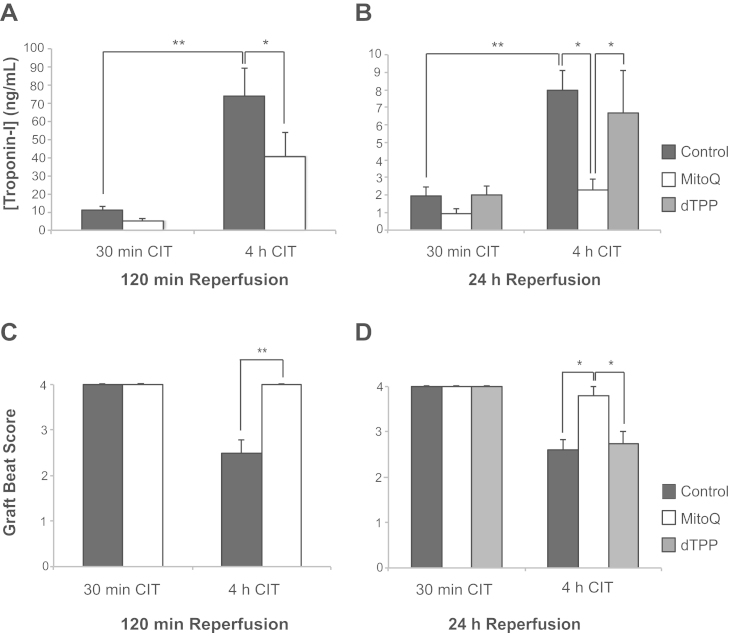
MitoQ protects against ischemia-reperfusion injury after heterotopic cardiac transplantation in mice. (A) Quantification of myocardial damage 120 minutes post-transplantation by serum troponin I after short (30 minutes) and prolonged (4 hours) cold ischemia time (CIT) preservation, with or without treatment with MitoQ (*n* = 4–5). (B) Quantification of myocardial damage 24 hours post-transplantation by serum troponin I after short (30 minutes) and prolonged (4 hours) cold preservation, with or without treatment with MitoQ or decyltriphenylphosphonium (dTPP; *n* = 4–5). Note the 10-fold decrease in the scale compared with panel A. Blinded assessment of heart function (C) 120 minutes and (D) 24 hours after transplant by beating score (*n* = 4–5). *p < 0.05, **p < 0.01, determined by 1-way analysis of variance with Bonferroni post-test. Data are mean ± standard error of the mean.

We chose a syngeneic transplant model to focus on mitochondrial events during post-transplant IR injury in the absence of an alloimmune response. We preserved donor hearts at 4°C for 30 minutes or 4 hours before heterotopic transplantation (Figure 1A). Transplantation after 4 hours of cold ischemia time (CIT) resulted in greater graft injury than after 30 minutes of CIT, as indicated by serum troponin levels 2 hours and 24 hours after transplant (Figure 3A and B). Heart contractions were also weakened and arrhythmic after 4 hours of CIT compared with 30 minutes of CIT (Figure 3C and D).

Administration of MitoQ during 4 hours of cold preservation significantly decreased post-transplant graft damage, as evidenced by decreased serum troponin levels at 24 hours (Figure 3B). MitoQ also improved beat scores at 24 hours (Figure 3D). At 24 hours there was evidence of early histologic damage in the 4-hour cold preservation group, including focal myocyte damage and ballooning, but minimal cellular infiltration and no necrosis. As expected, the overall extent of histopathologic damage at this early time (24 hours) was low. The damage was reported less frequently in the group treated with MitoQ, but this was unlikely to be of functional consequence due to the general low level of damage. The differences were therefore not quantified. Treatment of donor grafts with the control compound dTPP, which contains the mitochondria-targeting TPP lipophilic cation without the ubiquinol anti-oxidant so will still be accumulated by mitochondria but will not prevent oxidative damage, did not reduce serum troponin levels (Figure 3B) or improve the graft beat score (Figure 3D).

### Prolonged CIT increases graft mitochondrial ROS production and oxidative damage

**Figure 4:**
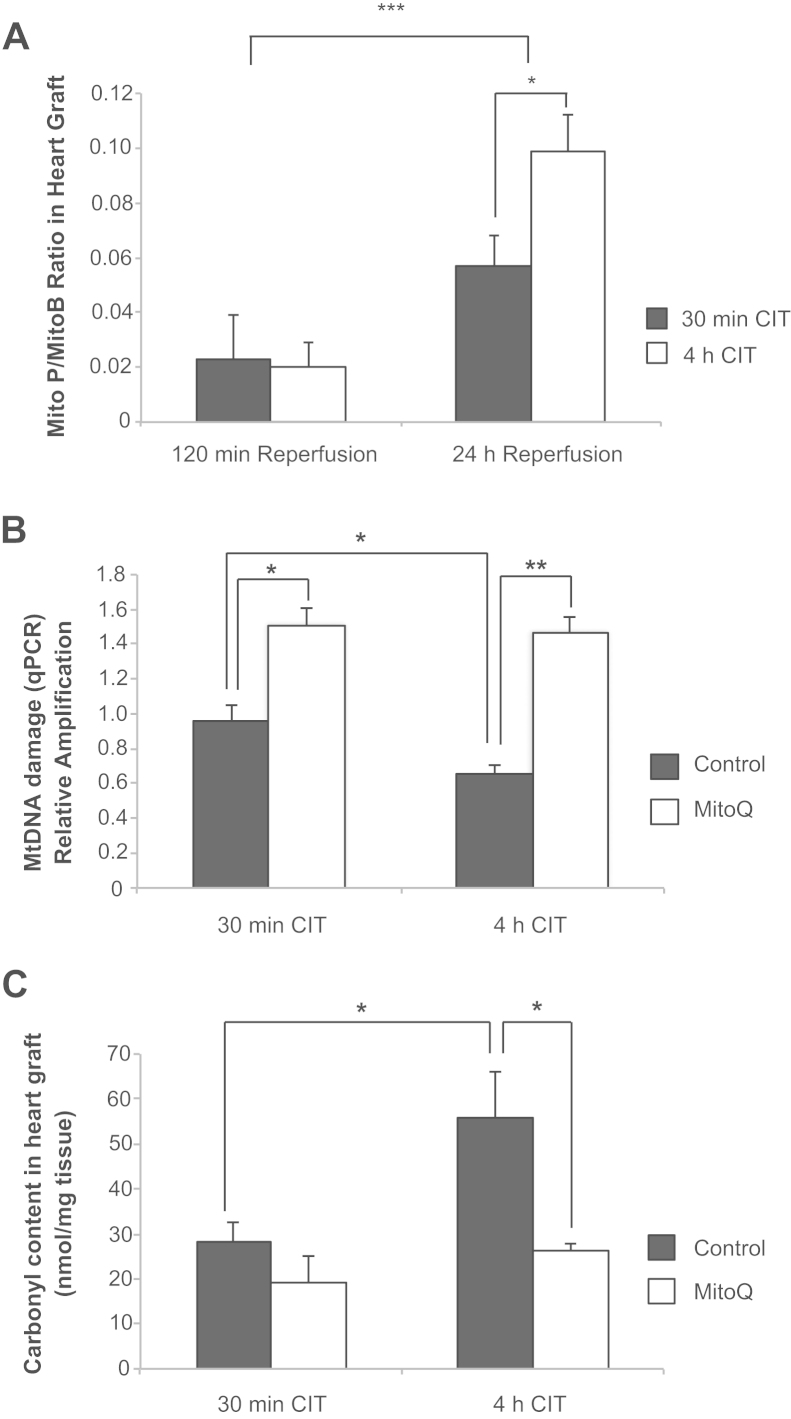
Prolonged cold preservation results in increased mitochondrial reactive oxygen species (ROS) and oxidative damage within the graft 24 hours after transplant, and MitoQ reduces this oxidative damage. (A) The MitoP/MitoB ratio, which increases in response to elevated mitochondrial H_2_O_2_, was measured within the heart graft in vivo at 24 hours post-transplant after short and prolonged cold ischemia time (CIT) preservation (*n* = 4). (B) Mitochondrial DNA (mtDNA) damage in the heart graft 24 hours after transplantation. Decreased mtDNA amplification is detected by quantitative polymerase chain reaction (qPCR) using a mitochondrial long target relative to a short target, which reflects damage. mtDNA amplification levels from heart grafts exposed to short or prolonged cold preservation, with or without MitoQ, have been normalized to healthy control mouse heart mtDNA amplification (*n* = 4). (C) Assessment of oxidative damage to proteins measured by protein carbonyl formation in heart grafts 24 hours after transplantation (*n* = 3–4). *p < 0.05, **p < 0.01, ***p < 0.001 determined by 1-way analysis of variance. Data are mean ± standard error of the mean.

We hypothesized that MitoQ protects against mitochondrial oxidative damage caused by mitochondrial ROS, preventing clinically relevant myocardial damage and graft dysfunction, especially in grafts exposed to prolonged cold preservation. Therefore, we next assessed the effect of CIT duration on mitochondrial ROS production and oxidative damage. To assess changes in production of the ROS H_2_O_2_ and peroxynitrite by mitochondria, we used the mitochondria-targeted ratiometric probe MitoB (Figure 4A). Measurement of mitochondrial ROS by this assay early after transplantation (0–2 hours; Figure 4A) showed no effect of CIT, whereas at 22 to 24 hours after transplantation, there was a significant increase in mitochondrial ROS production on extending CIT from 30 minutes to 4 hours (Figure 4A).

To determine whether mitochondrial oxidative damage also increased with CIT, we measured mtDNA damage by using a quantitative polymerase chain reaction assay and protein carbonyl formation (a marker of protein oxidative damage). mtDNA damage was seen in all groups 24 hours after transplantation compared with control mouse heart tissue, suggesting that an increase in mitochondrial oxidative damage is inherent in transplantation. Importantly, this oxidative damage was greater after 4 hours of CIT compared with 30 minutes and was decreased by MitoQ (Figure 4B).

Protein carbonyls accumulated in the heart 24 hours post-transplant and grafts transplanted after 4 hours of CIT had higher protein oxidative damage than those exposed to 30 minutes of CIT. MitoQ significantly decreased protein carbonyl formation in the 4-hour group but not in the 30-minute group (Figure 4C). Together these findings indicate that increased duration of CIT increases mitochondrial oxidative damage to the graft and that MitoQ decreases this damage.

### Prolonged CIT augments the inflammatory response

**Figure 5:**
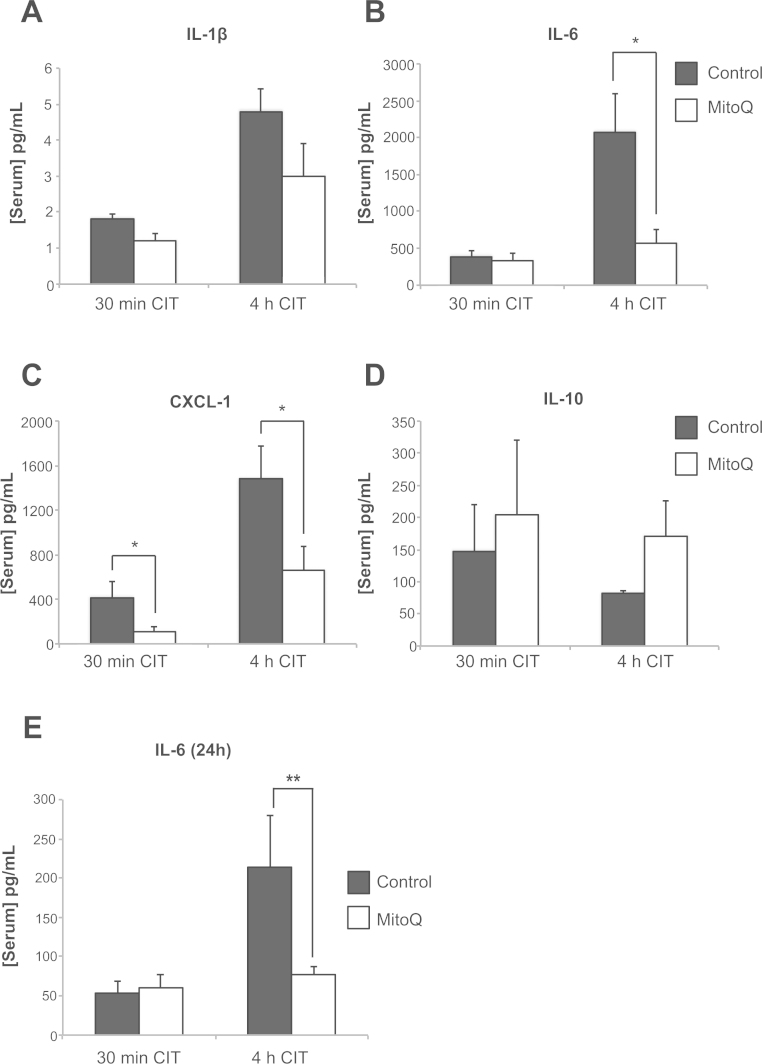
Prolonged cold preservation increases the systemic pro-inflammatory response after transplantation, which is ameliorated by MitoQ. (A) After 120 minutes of reperfusion there was a significant increase in interleukin (IL)-1β from 30 minutes to 4 hours of cold ischemia time (CIT) preservation (*p* = 0.004, 1-way analysis of variance [ANOVA]); however, treatment with MitoQ did not significantly reduce IL-1β (not significant, Bonferroni post-testing). (B) After 120 minutes there was a significant difference increase in IL-6 from 30 minutes to 4 h CIT (*p* < 0.05, Bonferroni post-testing). Treatment with MitoQ significantly reduced serum IL-6 for 4 h CIT (*p* < 0.05, Bonferroni post-testing). (C) After 120 minutes of reperfusion, there was a significant increase in chemokine (C-X-C motif) ligand 1 (CXCL-1) from 30 minutes to 4 hours CIT (*p* = 0.006, 1-way ANOVA). Treatment with MitoQ during 30 minutes of CIT and 4 hours of CIT significantly reduced serum CXCL-1 (*p* < 0.05, Bonferroni post-testing). (D) There were no significant differences in the levels of the anti-inflammatory cytokine IL-10 at 120 minutes of reperfusion with CIT or MitoQ treatment (not significant, 1-way ANOVA). (E) At 24 hours of reperfusion, serum IL-6 remained significantly elevated in the 4-hour CIT control group compared with 30 minutes of CIT (*p* < 0.01, 1-way ANOVA, Bonferroni post-testing), whereas serum IL-1β and CXCL-1 had normalized in all groups at 24 hours after reperfusion (data not shown). Treatment with MitoQ prevented the increase in IL-6 caused by 4 hours of CIT when measured 24 hours after reperfusion (*p* < 0.01, 1-way ANOVA, Bonferroni post-testing). For all experiments *n* = 4–5. *p < 0.05, **p < 0.01. Data are mean ± standard error of the mean.

Oxidative damage to the graft activates the recipient innate immune response through sterile inflammation.^35^ The increased mitochondrial oxidative damage during prolonged cold preservation was associated with a significant increase in serum levels of the pro-inflammatory cytokines IL-1β, CXCL-1, and IL-6 at 120 minutes after transplant (Figure 5A–C) but not in the anti-inflammatory cytokine IL-10 (Figure 5D). At 24 hours after transplantation, IL-6 was still significantly raised in recipients of grafts that had undergone prolonged cold preservation (Figure 5E), but IL-1β, CXCL-1 and IL-10 had normalized (data not shown). Tumor necrosis factor-α was unchanged in all groups (data not shown). MitoQ attenuated circulating CXCL-1 and IL-6 in the group with prolonged cold preservation. At 24 hours after transplant, IL-6 levels were significantly attenuated in grafts exposed to 4 hours of CIT with MitoQ (Figure 5E). Therefore, the damage sustained by the graft leads to early activation of a systemic pro-inflammatory response that corresponds to the degree of graft injury.

## Discussion

IR injury increases morbidity and mortality from early graft dysfunction and limits the ability to expand organ preservation periods and, therefore, the organ donor pool. Here, we have shown that the mitochondria-targeted anti-oxidant MitoQ accumulates within the cardiac graft during cold preservation and protects against post-transplant dysfunction by reducing oxidative damage and inflammation. Because the focus of this study was to assess whether adding MitoQ to the flush solution was effective in preventing oxidative damage during transplantation, the assessment of heart function was imprecise, and future work will return to a more detailed assessment of the preservation of function in the beating heart. These findings provide further evidence of the importance of mitochondrial oxidative damage in the pathogenesis of graft dysfunction, particularly after prolonged CIT where graft injury is most pronounced, and provide proof-of-principle evidence to support further investigation of the therapeutic use of mitochondria-targeted anti-oxidants in organ transplantation.

Prolonged cold preservation of human donor hearts is associated with increased IR injury and primary graft dysfunction after transplant.^5^ Previous studies of heterotopic cardiac transplantation in rodents have demonstrated that prolonged cold preservation worsens graft function.^35^, ^36^, ^37^, ^38^, ^39^, ^40^ However, none of these studies established a clear role for mitochondrial oxidative damage. Using MitoB, we were able to demonstrate that prolonged cold preservation of donor hearts increases mitochondrial ROS generation in the heart graft 24 hours after transplant. This was associated with increased oxidative damage to protein and mtDNA in the transplanted graft and heightened IR injury. Treatment of donor hearts with MitoQ during organ preservation significantly reduced oxidative damage and was associated with decreased heart damage and improved contractility after transplant.

We deliberately chose a syngeneic transplant model to first elucidate the mechanistic role of mitochondria in post-transplant IR injury in the absence of an alloimmune response. In this syngeneic model, prolonged CIT resulted in a heightened systemic inflammatory response as evident by the increase in serum levels of pro-inflammatory cytokines. Consistent with our findings, mitochondrial damage is known to contribute to the initiation of sterile inflammation and innate immunity in a variety of pathologic settings. One mechanism is through the release of mitochondrial components from damaged cells that act as mitochondrial damage-associated molecular patterns (mtDAMPs) such as oxidized mtDNA.^41^ These activate inflammasomes (especially NACHT, LRR and PYD domains-containing protein 3 [NLRP3]) and Toll-like receptor 9. Inflammasome activation leads to the release of the pro-inflammatory cytokine IL-1β, which then activates downstream cytokines, including IL-6, known to be associated with graft dysfunction after transplantation.^13^, ^42^, ^43^, ^44^, ^45^ Recipients of grafts exposed to prolonged cold ischemia showed significantly higher circulating levels of IL-6 and also other pro-inflammatory cytokines (IL-1β, CXCL-1). MitoQ treatment of the donor hearts lowered circulating IL-6 and CXCL-1 levels, suggesting that MitoQ decreased the pro-inflammatory response to the graft by decreasing mitochondrial oxidative damage and subsequent mtDAMP release. This is important, because in an allogeneic transplant, a heightened innate immune response to tissue injury may exacerbate the alloimmune response to the engrafted tissue, which is associated with chronic rejection.^46^

Several organ-preservation solutions in clinical use contain untargeted anti-oxidants, but their role in mitigating primary graft dysfunction remains unproven. For example, glutathione is added to University of Wisconsin solution during its commercial preparation but rapidly oxidizes to glutathione disulfide in solution, abrogating any protective effect at reperfusion.^47^ Furthermore, conventional untargeted anti-oxidants have typically produced disappointing clinical results because they are not taken up into the mitochondria in vivo.

Confirmation of MitoQ uptake into the graft during cold preservation, along with its therapeutic efficacy in reducing post-transplant IR injury, suggests that mitochondria-targeted anti-oxidants are a promising new therapeutic approach for use in transplantation. An oral preparation of MitoQ has already safely undergone Phase I and II clinical trials.^23^, ^24^ However, we note that no efficacy was shown in one trial^23^ and that the efficacy shown in the other trial was small.^24^ Therefore, although MitoQ has the potential for translation to clinical transplantation further, studies in large-animal models will clearly be required.

**Figure 6:**
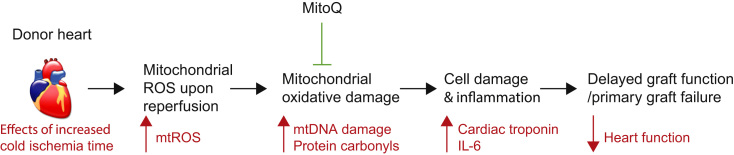
This schematic summarizes the conclusions from this work. Increasing the duration of cold ischemia exacerbates the production of mitochondrial reactive oxygen species (mtROS) upon reperfusion of the donor heart within the recipient. This ROS production leads to mitochondrial oxidative damage that in turn causes the cell death and inflammation that underlies delayed graft function and primary graft failure. Infusing the mitochondria-targeted anti-oxidant MitoQ into the donor heart before storage decreases this oxidative damage and the subsequent inflammatory response. IL, interleukin; mtDNA, mitochondrial DNA.

Together, our data show that increased mitochondrial oxidative damage is a major contributor to poor graft outcome in the early post-transplant period and that this can be prevented by MitoQ (Figure 6). Because a critical determinant of the extent of oxidative damage to the graft was the length of cold ischemia, this suggests that increased CIT may be disruptive because it increases the extent of mitochondrial oxidative damage upon reperfusion of the graft. This oxidative damage to mitochondria causes immediate tissue injury and heart dysfunction while also activating the innate immune response through the production of mtDAMPs.^41^ Treatment of the graft with MitoQ prevented the initial damage and the activation of the inflammatory response. These findings suggest that mitochondria-targeted therapies designed to minimize mitochondrial oxidative damage may decrease post-transplant graft dysfunction. This offers the potential to improve current outcomes after cardiac transplantation and to expand the pool of grafts suitable for transplantation.

## Disclosure statement

This work was supported by the Medical Research Council and National Institute for Health Research Cambridge Biomedical Research Centre.

M.P.M. holds shares in a company developing MitoQ as a potential pharmaceutical. None of the other authors has a financial relationship with a commercial entity that has an interest in the subject of the presented manuscript or other conflicts of interest to disclose.
