# Supplementary material for: The mitochondria-targeted anti-oxidant MitoQ decreases ischemia-reperfusion injury in a murine syngeneic heart transplant model
Source: J Heart Lung Transplant. 2015 Nov;34(11):1471–80. doi: 10.1016/j.healun.2015.05.007 (PMC4626443; doi:10.1016/j.healun.2015.05.007)
Supplement: Supplementary file 1 — Supplementary Material [file mmc1.docx]

**Supplementary Methods**

**MitoQ and dTPP**

MitoQ [10-(4,5-dimethoxy-2-methyl-3,6-dioxo-1,4-cyclohexadien-1-yl)decyl]triphenylphosphonium methanesulfonate, complexed with β-cyclodextrin, was supplied by Antipodean Pharmaceuticals. Decyltriphenylphosphonium (dTPP) bromide,^1^ was complexed with β-cyclodextrin. The flush and preservation solutions were supplemented with 50 µM MitoQ or dTPP.

**Uptake of TPMP by Isolated Mitochondria**

To measure accumulation of methyltriphenyl phosphonium (TPMP), rat liver mitochondria, prepared by homogenisation followed by differential centrifugation, were incubated at 2 mg protein/mL at 37˚C or 4˚C ± 500 nM *p*-trifluoromethoxyphenylhydrazone (FCCP) for 5 min in 240 µL medium (120 mM KCl, 10 mM HEPES, pH 7.2 and 1 mM EGTA), supplemented with 10 mM succinate, 4 µg/mL rotenone and TPMP (500 nM) supplemented with [^3^H]-TPMP (100 nCi/mL, American Radiolabelled Chemicals, St Louis, MO). The mitochondria were then pelleted by centrifugation (14,000 x *g* for 5 min) and the [^3^H]-TPMP content of supernatants and pellets were determined by scintillation counting.^1^ The accumulation ratios (ACRs) (TPMP per mg mitochondrial protein)/ TPMP per µL of supernatant) are given without calculating the membrane potential as temperature changes may alter volume or TPMP membrane binding.

**Measurement of MitoQ Tissue Concentration**

MitoQ in donor hearts was measured by liquid chromatography tandem mass spectrometry (LC-MS/MS) relative to a deuterated internal standard (*d_15_*-MitoQ)^2^. Donor hearts were briefly rinsed in saline to remove MitoQ from the organ surface and then flash frozen in liquid nitrogen. To extract MitoQ, heart tissue (20 mg wet weight) was homogenised in a 2 mL eppendorf tube at 4 °C using a bullet blender (Next Advance Inc, NY) in 500 µL Tris-HCl buffer (50 mM, pH 7.0), spiked with internal standard (50 pmol *d_15_*-MitoQ), vortexed (10 s) and 1.5 mL 95% acetonitrile (ACN)/ 0.1% formic acid (FA) added and vortexed again and centrifuged (10 min at 16,000 *x g*). The supernatant was transferred to a fresh tube, the pellet re-extracted and the combined supernatants dried under vacuum (Savant SpeedVac 3-4 h). The dried sample was resuspended in 200 µL 20% ACN/0.1% FA, vortexed (5 min), sonicated (Ultra BT Ultrasonic Bath, Ultrawave Ltd) (5 min), centrifuged (10 min at 16,000 *x g*) and filtered (0.22 µm PVDF filter, Millex from Millipore) into an autosampler vial (1.5 mL silanised, Chromacol). Samples were then analysed by LC-MS/MS with multiple reaction monitoring (MRM) in positive ion mode using a Xevo TQ-S mass spectrometer and an I-Class Acquity UPLC (both from Waters, UK). Data was analysed using MassLynx MS software (Waters, UK). A standard curve was prepared in parallel using tissue spiked with *d_15_*-MitoQ and different concentrations of MitoQ.

**Determination of Relative mtDNA Damage**

To assess mtDNA damage, total DNA was isolated from frozen heart graft tissue (~20 mg wet weight) using the Qiagen DNeasy Tissue Kit (Qiagen, UK) and quantified using the PicoGreen dsDNA Assay Kit (Invitrogen). Damage to mtDNA was then assessed using a quantitative PCR method.^3^ Each sample was corrected for the non-template control, amplification of the long PCR target was normalized to that of the short target and amplification relative to heart mtDNA from sham-operated controls was calculated for each sample to indicate mtDNA damage.

**Measurement of Protein Carbonyl Formation**

Total protein carbonyl concentration in heart tissue was determined by ELISA using the BioCell PC test kit (Biocell Corp, Auckland, New Zealand).^4^ Tissue samples (50 mg wet weight) were homogenized in PBS supplemented with a protease inhibitor cocktail (Complete, Mini, EDTA-free tabs, Roche) at 4 °C ^5^ and the supernatant collected and protein quantified relative to a bovine serum albumin standard (BCA Protein Assay kit, Pierce). Following derivation with dinitrophenylhydrazine, carbonyl determination in samples and standards was measured using an ELISA method according to the manufacturer’s instructions.

**References:**

^1^ Ross MF, Prime TA, Abakumova I *et al*. Rapid and extensive uptake and activation of hydrophobic triphenylphosphonium cations within cells. *Biochem J.* 2008;411:633-645

^2^ Rodriguez-Cuenca S, Cochemé HM, Logan A *et al*. Consequences of long-term oral administration of the mitochondria-targeted antioxidant MitoQ to wild-type mice. *Free Rad Biol Med* 2010; 48: 161-172.

^3^ Santos JH, Meyer JN, Mandavilli BS, Van Houten B. Quantitative PCR-based measurement of nuclear and mitochondrial DNA damage and repair in mammalian cells. *Meth Mol Biol.*  2006:314;183-199

^4^ Winterbourn CC, Buss IH. Protein carbonyl measurement by enzyme-linked immunosorbent assay. *Methods Enzymol.* 1999;300:106-111.

^5^ Vanderlelie JH, Venardos K, Clifton VL, Gude NM, Clarke FM, Perkins AV. Increased biological oxidation and reduced antioxidant activity in pre-eclamptic placentae. *Placenta*. 2005;26:53-58.
